# Absence of evidence for Palaeoproterozoic eclogite-facies metamorphism in East Antarctica: no record of subduction orogenesis during Nuna development

**DOI:** 10.1038/s41598-021-86184-4

**Published:** 2021-03-24

**Authors:** Dillon A. Brown, Laura J. Morrissey, John W. Goodge, Martin Hand

**Affiliations:** 1 Department of Earth Sciences, University of Adelaide, Adelaide, SA Australia; 2 Future Industries Institute, University of South Australia, Mawson Lakes, SA Australia; 3 Department of Earth and Environmental Sciences, University of Minnesota, Duluth, MN USA

**Keywords:** Precambrian geology, Petrology, Tectonics

## Abstract

The cratonic elements of proto-Australia, East Antarctica, and Laurentia constitute the nucleus of the Palaeo-Mesoproterozoic supercontinent Nuna, with the eastern margin of the Mawson Continent (South Australia and East Antarctica) positioned adjacent to the western margin of Laurentia. Such reconstructions of Nuna fundamentally rely on palaeomagnetic and geological evidence. In the geological record, eclogite-facies rocks are irrefutable indicators of subduction and collisional orogenesis, yet occurrences of eclogites in the ancient Earth (> 1.5 Ga) are rare. Models for Palaeoproterozoic amalgamation between Australia, East Antarctica, and Laurentia are based in part on an interpretation that eclogite-facies metamorphism and, therefore, collisional orogenesis, occurred in the Nimrod Complex of the central Transantarctic Mountains at c. 1.7 Ga. However, new zircon petrochronological data from relict eclogite preserved in the Nimrod Complex indicate that high-pressure metamorphism did not occur in the Palaeoproterozoic, but instead occurred during early Palaeozoic Ross orogenesis along the active convergent margin of East Gondwana. Relict c. 1.7 Ga zircons from the eclogites have trace-element characteristics reflecting the original igneous precursor, thereby casting doubt on evidence for a Palaeoproterozoic convergent plate boundary along the current eastern margin of the Mawson Continent. Therefore, rather than a Palaeoproterozoic (c. 1.7 Ga) history involving subduction-related continental collision, a pattern of crustal shortening, magmatism, and high thermal gradient metamorphism connected cratons in Australia, East Antarctica, and western Laurentia at that time, leading eventually to amalgamation of Nuna at c. 1.6 Ga.

## Introduction

An understanding of the Earth’s record of collisional orogenesis is required to assemble past supercontinents^1–4^. Evidence for the existence of the ancient supercontinent Nuna is based in part on the occurrence of 2.1–1.8 Ga collisional orogenic belts in the geological record as well as paleomagnetic constraints^1^. Although the timing of Nuna amalgamation is debated, with competing estimates of either 1600–1500 Ma^4–9^ or c. 1740 Ma^3,10–12^, the most recent reconstructions position the Mawson Continent (South Australian Craton and East Antarctica) and the North Australian Craton adjacent to the western Laurentian margin in an incipient southwest United States–East Antarctica (proto-SWEAT) configuration^4,5,9^.

**Figure 1:**
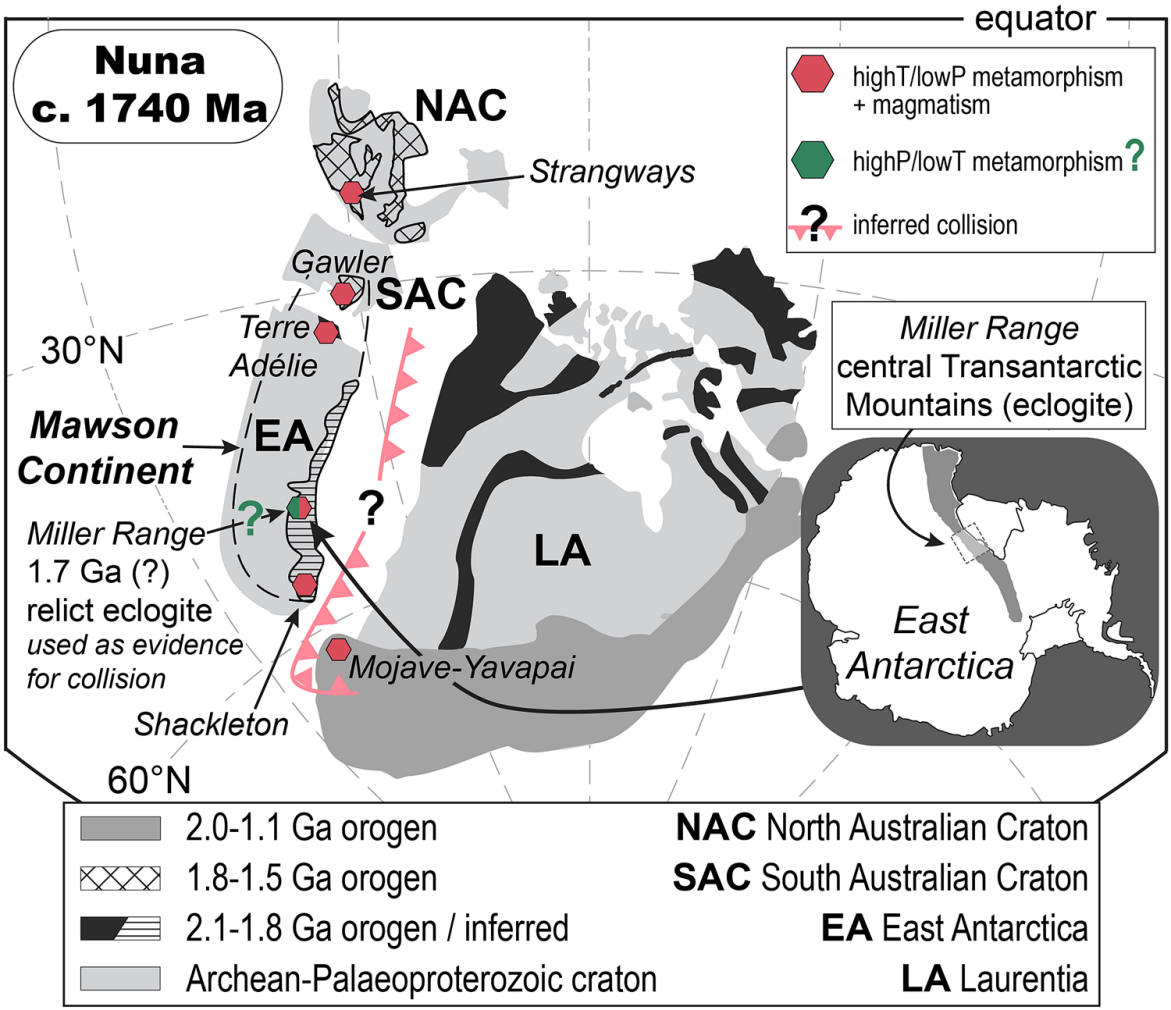
Palaeogeographic reconstruction of Nuna modified after Zhang et al.^3^, Betts et al.^5^, and Goodge et al.^21^. Modified using Adobe Illustrator (v. 24.3, https://www.adobe.com/au/products/illustrator.html)^22^. The approximate boundary of the Mawson Continent is shown with a dashed line^23^. The modern position of the Miller Range is shown by the inset map. The palaeogeographic locations of the Mojave and Yavapai provinces, Shackleton Range, Miller Range, Terre Aélie, Gawler Craton and Strangways Orogen show the occurrences of Palaeoproterozoic metamorphism and magmatism in these regions, collectively used to link sites of orogenesis and to infer a Palaeoproterozoic plate boundary position for the Miller Range adjacent to Laurentia. The timing of active plate-margin orogenesis and amalgamation of Australia/East Antarctica and western Laurentia is debated, with assembly by c. 1740 Ma^3,10–12^ or 1600–1500 Ma^4–9^. The previously interpreted occurrence of c. 1720 Ma eclogite-facies metamorphism in the Miller Range has been used as an indicator of plate-margin orogenesis between Australia/East Antarctica, and western Laurentia, consistent with the former model for amalgamation.

A key geological constraint underpinning Nuna reconstructions is the documentation of contemporaneous 1730–1720 Ma orogenic events in South Australia’s Gawler Craton and in the central Transantarctic Mountains of East Antarctica, which appear to link the Palaeoproterozoic Kimban Orogeny in South Australia and the Nimrod Orogeny in East Antarctica^10,11,13–15^. This led to the suggestion of a continuous 1730–1690 Ma collision-subduction margin connecting these two regions and defining the eastern active margin of the Mawson Continent^11^ (Fig. 1), which may also extend into the Mojave Province in Laurentia as the Ivanpah Orogeny^13,16^. The interpretation of a collisional Mawson margin hinges on the occurrence of Palaeoproterozoic eclogite-facies rocks in the Miller Range of the central Transantarctic Mountains (Nimrod Complex)^13,17^. The importance of eclogite-facies rocks within Nuna reconstructions was recently emphasized by Wan et al.^18^, who identified a network of sutures related to the assembly of Nuna that are marked by the preservation of 2.0–1.8 Ga eclogites. Eclogites are crucial markers of subduction-style orogenic processes^19^ and the c. 1720 Ma eclogites from the Miller Range have been used as a cornerstone in the interpretation that the current eastern margin of the Mawson Continent was a Palaeoproterozoic convergent plate boundary adjacent to western Laurentia (Fig. 1). However, recent re-examination of the Miller Range eclogites has shown that these rocks experienced conditions of 16–18 kbar and 675–760 °C during the Cambro-Ordovician Ross Orogeny^20^, casting doubt on the evidence for high-pressure metamorphism in the Paleoproterozoic.

Here, we present new petrochronological data from a second, previously studied Palaeoproterozoic eclogite from the Miller Range showing that the Palaeoproterozoic-aged zircons did not form during high-pressure metamorphism. These new findings remove critical evidence used to infer c. 1.7 Ga subduction and convergence along the margin of the Mawson Continent bounded by the present-day Cambro-Ordovician Ross Orogen. In contrast, the Palaeoproterozoic record from the Nimrod Complex is in accord with other evidence of c. 1.7 Ga low-pressure, high-temperature metamorphism and magmatism extending from the North Australian Craton to the South Australian Craton, East Antarctica, and western Laurentia^24–28^. Our findings thus highlight the importance of using trace-element compositions in zircon to distinguish eclogite-facies from igneous protolith signatures in order to evaluate the global Palaeoproterozoic record of high-pressure metamorphism and to determine the timing of amalgamation between crustal elements within supercontinent reconstructions.

## Significance of the Miller Range relict eclogite

**Figure 2:**
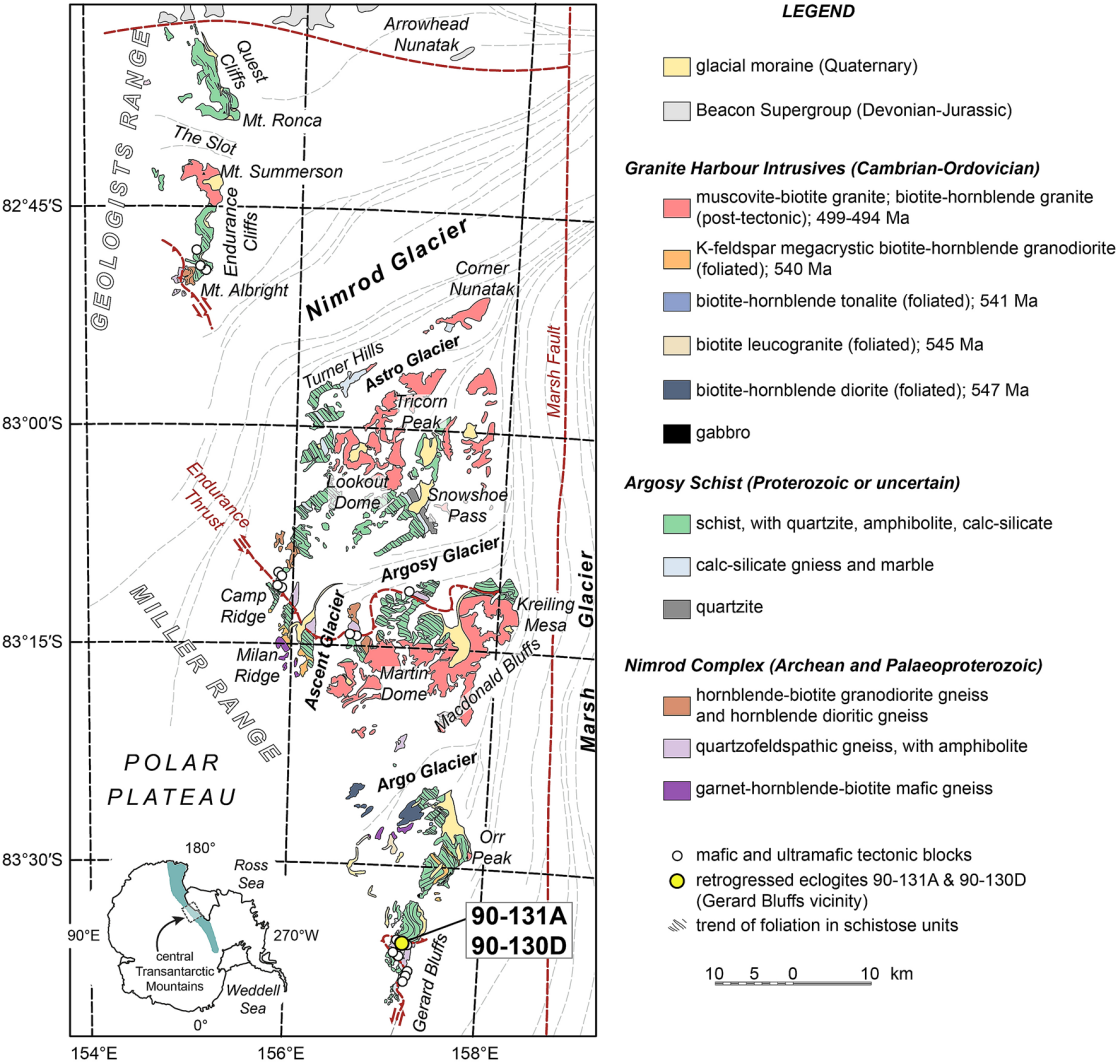
Geological map of the Miller Range, central Transantarctic Mountains. The location of relict eclogite samples 90-131A (subject of this study) and 90-130D^20^ in the Nimrod Complex is given by a yellow circle (83°35′23″ S; 157°10′ E). Figure modified from Goodge and Fanning^23^ using Adobe Illustrator^22^. Note that the two eclogite samples come from different nearby outcrops, although they are shown at the same location at the scale of this map.

Eclogite-facies rocks are rare in the Palaeoproterozoic geological record^19,29^, but are critical in delineating the existence of palaeosubduction systems. Previous geochronology indicated that the Nimrod Complex in the Miller Range, located in the modern Transantarctic Mountains, records one such example of Palaeoproterozoic eclogite-facies metamorphism^13^. The Nimrod Complex contains a diverse assemblage of amphibolite- to granulite-facies quartzofeldspathic, migmatitic, mafic and granitic gneisses, that enclose boudinaged mafic blocks preserving relict eclogite-facies mineral assemblages^17,23^ (Fig. 2). Although the Nimrod Complex rocks have been pervasively overprinted by the Neoproterozoic to early Paleozoic Ross Orogeny^30,31^, the existing geochronology highlights a record of Mesoarchean to Palaeoproterozoic magmatism and metamorphism^13,23,32,33^. SHRIMP U–Pb zircon ages of c. 1720 Ma from a relict eclogite^13^ (sample 90-131A) were interpreted to reflect Palaeoproterozoic eclogite-facies metamorphism and orogenesis related to crustal thickening driven by plate convergence and/or collision (Nimrod Orogeny). However, the U–Pb age data were not accompanied by zircon trace-element compositions that link the zircon ages explicitly to the high-pressure mineral assemblage. Applying in-situ petrochronological techniques to zircons from a second relict eclogite sample (90-130D) located in the same area as sample 90-131A (Gerard Bluffs, Miller Range; Fig. 2), Brown et al.^20^ documented c. 535 Ma eclogite-facies metamorphism in the Miller Range. Relict eclogite samples 90-130D and 91-131A are mineralogically and texturally similar, containing porphyroblastic garnet surrounded by clinopyroxene-plagioclase symplectites and Na-Ca amphibole. Additionally, garnet in 90-130D contains inclusions of omphacite^13,17,20^. Importantly, zircon grains in equilibrium with the eclogite assemblage and coeval with rutile in 90-130D have trace-element compositions similar to those in other eclogites, such as those studied by Rubatto et al.^34^

Whereas zircon ages from eclogite 90-131A are primarily Palaeoproterozoic, with only a small proportion showing slight isotopic resetting toward Cambrian ages^13^, the zircons in eclogite 90-130D are primarily Cambrian-aged, with a less dominant proportion giving Palaeoproterozoic ages which are highly discordant and typically significantly older than c. 1720 Ma^20^. Considering these contrasting age patterns between two mineralogically similar eclogitic samples from a similar location, and because the zircons from eclogite 90-131A lack trace-element data, it is necessary to obtain trace-element information from the Paleoproterozoic-aged zircons to establish whether: (1) the mafic eclogites of the Miller Range record two high-pressure events separated by ~ 1200 Myr, or (2) high-pressure metamorphism in the Miller Range was solely a consequence of active convergent margin processes along the Cambro-Ordovician East Gondwana margin^20^.

## Zircon petrochronology

**Figure 3:**
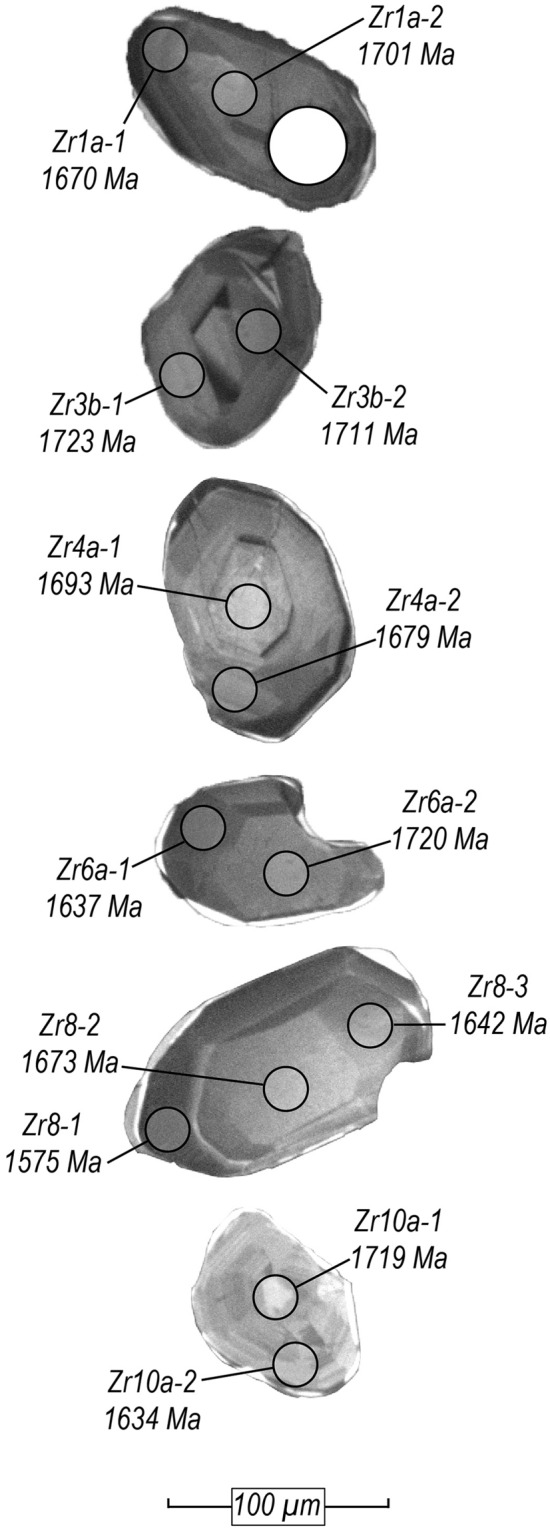
Cathodoluminescence images of representative zircons from sample 90-131A. Grey circles indicate the locations of 19 µm ablation pits. Spot ages are ^207^Pb/^206^Pb ages. The large white circle marks the location of a previous laser-ablation pit.

To test the previously proposed hypothesis that the Palaeoproterozoic zircons from sample 90-131A formed during high-pressure metamorphism along a plate boundary adjacent to western Laurentia, U, Pb, Th and trace-element isotopic concentrations were measured from twenty-three zircons using laser-ablation inductively-coupled mass spectrometry (LA–ICP–MS). Zircon U–Pb ages and trace-element concentrations are given in Supplementary Table S1. Zircons were separated using traditional mineral separation techniques as outlined by Goodge et al.^13^ Zircon grains are approximately 100–150 µm in length and have equant to semi-prismatic morphologies (Fig. 3). The zircons exhibit features typically observed in both magmatic or metamorphic zircon, showing concentric growth zoning patterns, sector zoning and less commonly, oscillatory zoning (Fig. 3). A common feature in many of the zircon grains are thin (~ 5–10 µm wide) bright-CL rims which were too small to target using laser ablation (Fig. 3). However, previous SHRIMP analysis of similar zircons gave rim ages between 535 and 480 Ma^23,33^.

**Figure 4:**
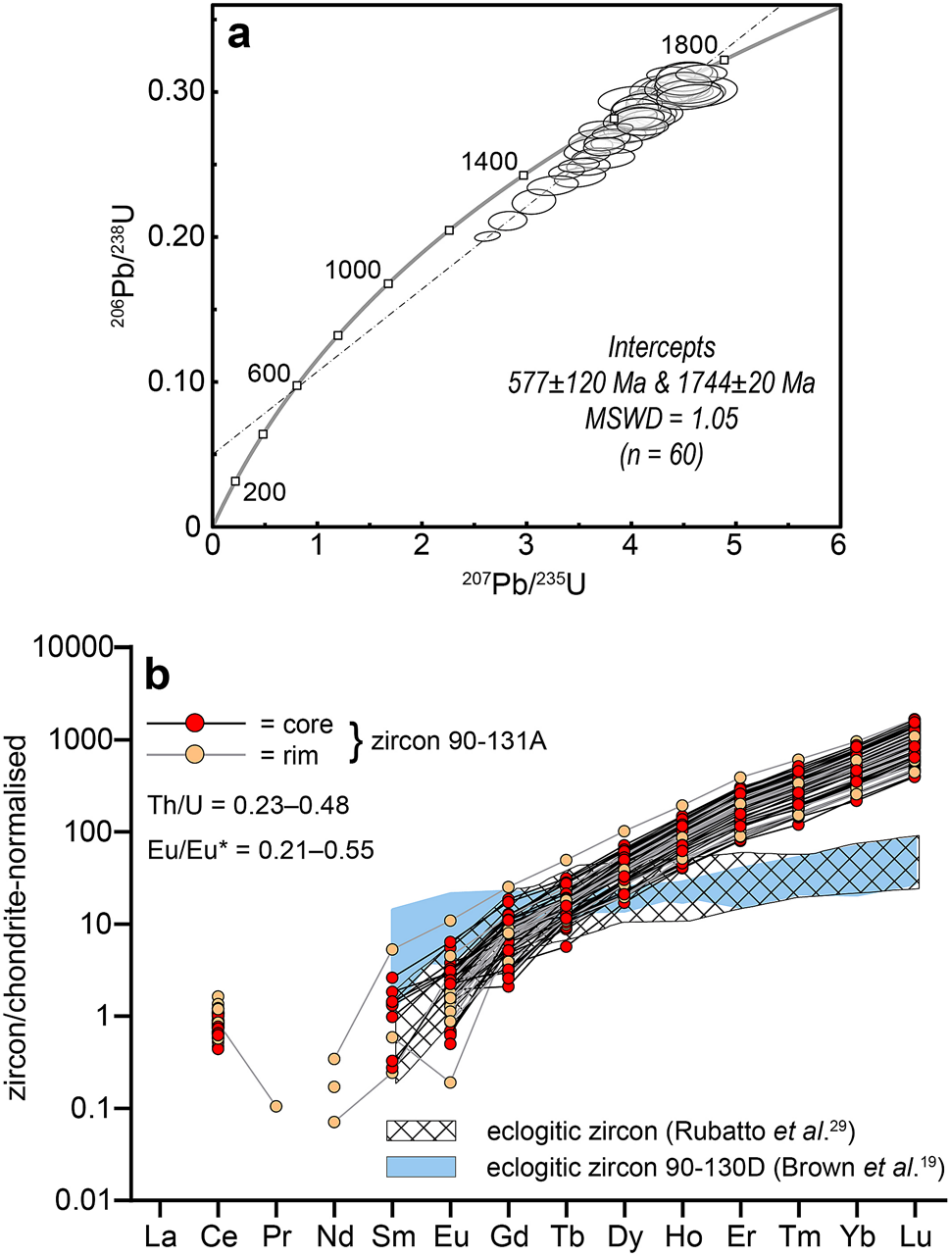
(**a**) Wetherill concordia plot showing U–Pb zircon analyses (all data, n = 60). 2σ uncertainties are at 95% confidence. (**b**) Chondrite-normalised rare earth element (REE) plot for 60 analyses of zircon. Normalisation values from Sun and McDonough^36^. Representative eclogitic zircon REE pattern is from eclogitic micaschist MST2a^34^ (Sesia–Lanzo Zone), and patterns obtained from zircons in eclogite sample 90-130D shown in blue^20^.

Sixty U–Pb analyses define a tight discordant array on a Wetherill concordia plot, with 11 analyses > 5% discordant (Fig. 4a). A regression line through all analyses yields a constrained upper-intercept age of 1744 ± 20 Ma (MSWD = 1.05) and a poorly constrained lower-intercept age of 577 ± 120 Ma, confirming that this sample records both the Nimrod and Ross orogenies. On a chondrite-normalised trace-element plot, all analyses have similar trace-element signatures, showing pronounced positively sloping heavy rare earth element (HREE) trends (Fig. 4b). There appears to be no correlation between trace-element concentration and internal zoning (Fig. 3b). Th/U ratios are between 0.23–0.48, consistent with values for magmatic zircon. Sm concentrations (in ppm) are below detection for most of the analyses; however, where Sm was detected, Eu anomalies (Eu*) are weakly negative (Supplementary Table S1). The magnitudes of the Eu anomalies (Eu/Eu*) range from 0.21 to 0.55 and are consistent with low-pressure zircon formation^35^ (Fig. 4b; Supplementary Table S1).

## Discussion

### Palaeoproterozoic or Cambrian high-pressure metamorphism in the Miller Range?

The new U–Pb age results from zircons in eclogite 90-131A are consistent with existing SHRIMP U–Pb data, yielding a Palaeoproterozoic upper-intercept age of 1744 ± 20 Ma, which is within uncertainty of the earlier c. 1723 ± 29 Ma intercept age^13^. Both datasets show Pb-loss trends projecting to Cambrian-age lower intercepts, attesting to overprinting during Ross-aged metamorphism. Goodge et al.^13^ interpreted the Palaeoproterozoic zircons from 90-131A to have formed during eclogite-facies metamorphism given that the zircons came from a rock containing an eclogite-facies mineral assemblage and the zoning features and morphologies pointed toward a possible metamorphic origin. The zircons were interpreted to have recrystallised texturally and structurally from igneous precursors. Moderate Th/U signatures (0.25–0.40), characteristic of igneous zircon, were thought to be partially retained from the igneous relicts.

However, our study shows that the trace-element compositions of these zircons are dissimilar to metamorphic zircons formed at high-pressure conditions, which typically have depleted chondrite-normalised HREE concentrations that reflect the presence of garnet^34,37–39^. Rather, the comparatively enriched HREE concentrations of these zircons are typical for zircons formed in a magmatic environment or during metamorphism at conditions which do not stabilize garnet^37,40,41^. Th/U ratios between 0.23–0.48 are in the range expected for igneous zircons and agree with those determined from SHRIMP analysis^13^, although this is not uniquely diagnostic of a magmatic paragenesis given the known variability in Th/U values in metamorphic zircon^40^, particularly in mafic rocks. Among those zircon analyses for which the Eu anomaly could be calculated, Eu anomalies are only slightly negative (Fig. 4b; Supplementary Table S1). The moderate Eu/Eu* values between 0.21–0.55 are typical of zircon in equilibrium with either magmatic or metamorphic plagioclase, the latter of which has a lower trace-element budget than magmatic plagioclase^34,35^. The textural morphologies of many of the zircons are consistent with a metamorphic origin (i.e., equant to ovoid, faceted ‘soccer-ball’ shapes), yet internal zoning features such as sector zones are consistent with both a magmatic and a high-grade metamorphic origin, and the presence of oscillatory zoning in some of the grains is suggestive of magmatic growth^40,42,43^ (Fig. 3). By re-examining the zircons in eclogite 90-131A, we can more confidently conclude from the new trace-element data that the zircons did not form in the presence of garnet at c. 1.7 Ga. Therefore, there is no evidence for eclogite-facies metamorphism at that time. The zircons are either magmatic in origin or formed during low-pressure metamorphism.

**Figure 5:**
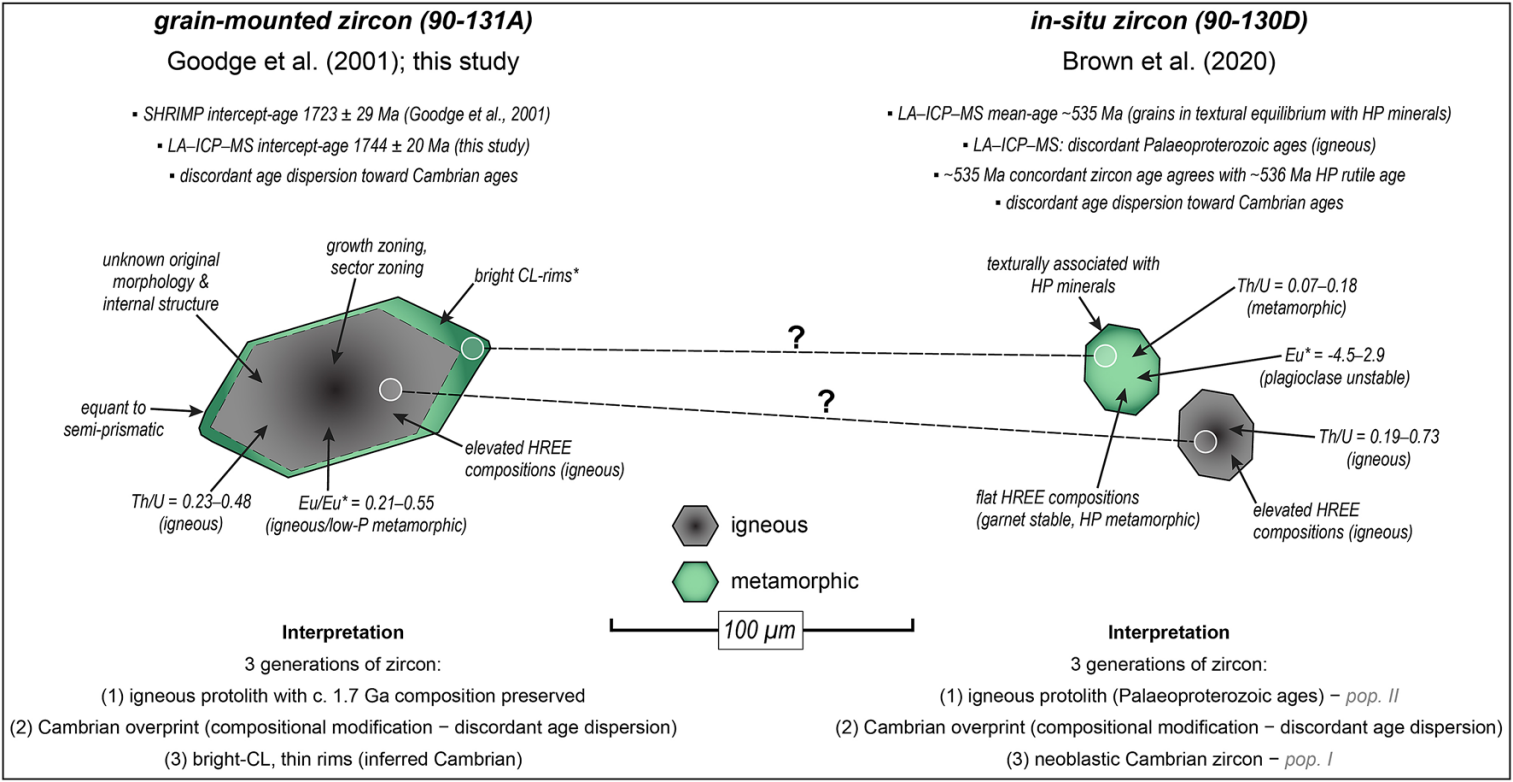
Schematic illustration comparing grain-mounted zircons from eclogite sample 90-131A (this study) and in-situ zircons from eclogite sample 90-130D^20^. Dashed lines connecting zircon domains infer that the domains may relate to a similar paragenesis. *Bright-CL rims have not been dated in this study, nor by Goodge et al.^13^. However, these rims are similar to low-Th zircon rims from Nimrod samples in the same area (gneissic samples), where the rims give U–Pb ages between 535 and 480 Ma^23,33^. For sample 90-130D, two different schemes were used by Brown et al.^20^ to define zircon types (populations I and II), which were assigned on the basis of composition. Zircon generations 1 and 3, here used to denote crystallisation sequence, are analogous to zircon populations II and I, respectively^20^.

Contrary to the earlier interpretation of Paleoproterozoic-aged eclogite-facies metamorphism in this sample^13^, we suggest that the zircons are primary igneous grains that have partially to completely retained their trace-element and isotopic characteristics through Cambro-Ordovician eclogite-facies metamorphism (Fig. 5). The primary morphologies and internal structures of the precursor igneous zircons are not known due to the effects of Ross-aged metamorphism and deformation, which likely promoted the development of new internal structures. Furthermore, it is conceivable that the ~ 5–10 µm wide-bright-CL rims on the igneous cores crystallised during Cambrian-aged high-pressure metamorphism, in agreement with previous SHRIMP ages of similar low-Th zircon rims from Nimrod gneissic samples (535–480 Ma)^23,33^. Zircon age and trace-element results coupled with mineral equilibria modelling from a nearby mafic sample indicate Cambro-Ordovician metamorphism was characterised by eclogite-facies conditions^20^. The differences in zircon ages and trace-element compositions between eclogite samples 90-131A (this study) and 90-130D^20^ can be reconciled with an interpretation that the Cambrian-aged neoblastic zircon grains in eclogite 90-130D are analogous to, and coeval with, the thin bright-CL rims characterising the igneous zircons from 90-131A (Fig. 5). It should be noted that no zircon grains having a c. 1.7 Ga age were found in sample 90-130D. This may be a consequence of the different procedures undertaken in determining the zircon ages (e.g., grain-mounted vs. in-situ), whereby the larger c. 1.7 Ga igneous relicts obtained by mineral separation were not found in polished thin-section, and the smaller zircons (in-situ) were not recovered through traditional mineral separation techniques. An alternative possibility is that the c. 1.7 Ga zircons from 90-131A were inherited from the Palaeoproterozoic gneisses enclosing the mafic eclogitic boudins, which is supported by extensively documented c. 1.7 Ga zircon ages from Nimrod rocks in the same area^23^. Given the absence of c. 1.7 Ga zircon ages in eclogite sample 90-130D^20^, it may be possible that c. 1.7 Ga zircon inheritance did not contribute to the zircon age dataset for this sample. A third possibility is that the eclogite precursors were petrologically similar mafic igneous rocks, possibly emplaced as dikes, but having different emplacement ages (90-130D yields a very poorly defined discordant array projecting to an upper-intercept at c. 2.2 Ga whereas 90-131A yields an upper-intercept age at c. 1.7 Ga). Of these three possibilities, the first is the simplest explanation given the data available, but we cannot discount the other explanations.

The results from this study, and other zircon age and trace-element patterns confirming an early Cambrian age for the Miller Range eclogite^20^, demonstrate that high-pressure metamorphism occurred as a consequence of plate convergence along the active East Gondwana margin during the early Palaeozoic Ross Orogeny. Therefore, an interpretation of c. 1.7 Ga subduction and high-pressure metamorphism along the eastern margin of the Mawson Continent is not supported by current evidence.

### Is there evidence for Palaeoproterozoic (1740–1690 Ma) collisional orogenesis between proto-Australia, the Mawson Continent, and Laurentia?

Multiple interpretations of the timing of amalgamation between Laurentia and the Mawson Continent have been proposed, with some workers advocating for assembly between c. 1740–1690 Ma^3,10–12^ and others preferring a more recent period between 1600 and 1500 Ma^4–9^. The older amalgamation model is supported by palaeomagnetic data and by Palaeoproterozoic igneous and metamorphic events recorded in East Antarctica (e.g., Terre Adélie, Miller Range, Shackleton Range), South Australia’s Gawler Craton, and western Laurentia (e.g., Mojave and Yavapai provinces), which have been temporally and petrologically correlated^23^ suggesting a tectonic linkage within Nuna. In this context, the earlier interpretation of Palaeoproterozoic eclogite-facies metamorphism in the Miller Range^13^ signified that the eastern margin of the Mawson Continent was a site of subduction-related collision and crustal thickening that potentially involved the western margin of Laurentia^4,11,15,23^ (Fig. 1). The Miller Range eclogites constituted the sole record of high-pressure metamorphism within a Nuna framework, otherwise dominated by Palaeoproterozoic (c. 1740–1690 Ma) metamorphism at low to moderate pressures and high temperatures recorded in Terre Adélie, the Shackleton Range, Gawler Craton, central Australia (Strangways Orogeny), and the Mojave Province^24–28^. Therefore, despite the lack of metamorphic evidence for Palaeoproterozoic subduction and/or collisional orogenesis in most of East Antarctica, South Australia, and western Laurentia^10^ (i.e., the absence of high-pressure metamorphic rocks signifying tectonic convergence and collision), metamorphism and magmatism in these regions is interpreted to have occurred as either a direct or indirect response to collision-subduction^11,15,23,44^ (Fig. 1).

However, the new results from zircon petrochronology indicate that the Palaeoproterozoic (c. 1720 Ma) Nimrod Orogeny in the central Transantarctic Mountains did not involve high-pressure metamorphism and subduction orogenesis. Instead, the Palaeoproterozoic record between 1740–1690 Ma is characterised by extensive magmatism and high thermal gradient metamorphism in Australia, East Antarctica, and western Laurentia^23–28,33^ (Fig. 1)—a record that is incompatible with subduction-related continental collision (e.g., Himalayan- or Caledonide-style collision). Without petrologic evidence for Palaeoproterozoic-aged subduction and collisional orogenesis between Australia/East Antarctica and western Laurentia, the thermal regimes recorded within these regions are quite similar, suggesting that a single coherent tectonothermal framework dominated by low-pressure metamorphism and crustal magmatism, and involving local tectonic deformation, may have existed at this time^23–28,33^.

### Relation to Nuna assembly

Based on the new data presented here and elsewhere^20^, a lack of evidence for c. 1.7 Ga eclogite-facies metamorphism in East Antarctica signifies that there was not a subduction-collisional regime in operation that was associated with Palaeoproterozoic-aged Nuna assembly between proto-Australia, the Mawson Continent, and western Laurentia. The lack of evidence for Palaeoproterozoic-aged high-pressure metamorphism in Australia, East Antarctica, and North America may simply reflect that eclogite-facies rocks in the respective metamorphic records were not preserved. Poor preservation of high-pressure rocks is not uncommon due to the mechanics of subduction and collision, as well as the potential for petrologic overprinting^19,45,46^, but the absence of Paleoproterozoic eclogites throughout this extensive region of Nuna raises question about the viability of tectonic models calling for subduction-collisional orogenesis among these cratonic elements.

Palaeogeographic and tectonic models of Nuna assembly are broadly defined as having occurred in one of three periods: (1) pre-1700 Ma, (2) c. 1800–1650 Ma, and (3) at c. 1650–1550 Ma. The c. 1.7 Ga events in proto-Australia, the Mawson Continent, and western Laurentia—notably widespread and not encompassing high-pressure metamorphism—help to inform Nuna assembly models. In the first case, the petrological and geochronological connections at c. 1.7 Ga within these key cratonic elements may indicate common crustal processes operating in an already-amalgamated Nuna. The shared patterns of low-pressure metamorphism and magmatism^23–28,33^ detailed earlier may have occurred within a broadly formed supercontinent. This model is supported by correlations between interpreted c. 1.85 Ga orogenies in northern Australia and north-west Laurentia (e.g., Barramundi and Trans-Hudson orogenies)^5,47^, and similarities between detrital zircons from rocks of the Mawson Continent and the Mojave Province^12^. However, there is no evidence for major inter-cratonic collision in these regions. Other models suggest progressive Nuna assembly by crustal thickening and accretionary processes active between approximately 1800–1600 Ma^3,11,15,44^. For example, Betts et al.^15^ correlated interior basins in proto-Australia and Laurentia, and propose that accretionary processes along the margins of Australia and southwestern Laurentia between 1800 and 1600 Ma extended from the margin of proto-Australia, through the Mawson continent, to Laurentia. As outlined earlier, these models are consistent with some palaeomagnetic data^3,48^ and evidence for Barrovian-style crustal thickening, thrust-style shortening, and accretionary imbrication from the magmatic, structural, and metamorphic records in Australia^10,15,25,49^, East Antarctica^23,24,28,33,50^, and North America^12,16,26,27,44,51,52^. Importantly, the evidence for Palaeoproterozoic moderate-pressure Barrovian-style metamorphism and crustal thickening does not imply the operation of collisional orogenesis as seen in the Himalayas, Alps, and Caledonides, all of which contain high- and ultrahigh-pressure eclogites^53–55^. However, these processes may signify the operation of broad-scale low-pressure, moderate- to high-temperature crustal interactions between proto-Australia, East Antarctica, and western Laurentia, potentially related to the early development of Nuna in the Palaeoproterozoic^5,48^. This interpretation may be explained by a setting involving a comparatively passive connection between the Mawson Continent and western Laurentia at this time, whereby a narrow marine basin may have existed between the cratonic fragments (see Kirscher et al.^9^ and references therein). Notably, the c. 1.7 Ga events recorded in proto-Australia, the Mawson Continent, and western Laurentia may be related to the ongoing processes described by these models more broadly, but they are not an indicator of subduction-collision at this time. Lastly, some models suggest that the cratonic elements within Nuna were amalgamated in a single collisional event during the Mesoproterozoic^9^, as suggested on the basis of petrological and geochronological investigations of garnet-bearing rocks from northern Australia^6,7,56^. Collision and assembly between proto-Australia and western Laurentia at c. 1.6 Ga^57^ is also suggested by Mesoproterozoic-aged (c. 1.65 Ga) Laurentian crust preserved in northern Australia^58^, 1.55–1.48 Ga post-orogenic activity recorded by thermochronological constraints and A-type granitoids in northern Australia^8^, and some palaeomagnetic data^4^. Continuation of the Mesoproterozoic northern Australian orogenic system into western Laurentia (e.g., Mazatzal and Racklan orogenies) is supported by Mesoproterozoic-aged tectonism preserved in both northern Australia and North America^59,60^. Despite clear evidence of c. 1.6 Ga tectonic activity, we do not favour the interpretation of a single Nuna amalgamation event in the Mesoproterozoic given: (1) an absence of evidence for c. 1.6 Ga subduction orogenesis and collision in the Australian and North American records, (2) the aforementioned evidence for Palaeoproterozoic (c. 1740–1690 Ma) Barrovian-style metamorphism and crustal thickening which is suggestive of tectonothermal activity related to early Nuna assembly, and (3) palaeomagnetic data indicating an earlier proximity between these elements^4,10,48^. As observed in the Palaeoproterozoic magmatic and metamorphic records, the Mesoproterozoic histories in Australia and North America are suggestive of high thermal gradient metamorphism and crustal thickening, particularly given the occurrence of staurolite- and andalusite-grade metamorphism in northern Australia^6,7^. Furthermore, evidence for c. 1.6 Ga felsic magmatism related to high-temperature processes is preserved in Terre Adélie^61^, the Gawler Craton^62^, and central East Antarctica^63^. Ultimately, the shared record of c. 1.7 Ga low-pressure metamorphism and magmatism in proto-Australia, the Mawson Continent, and western Laurentia, as well as from isotopically-similar 2.01–1.85 Ga igneous rocks, suggests a long-standing association that is inconsistent with initial Nuna assembly at 1.6 Ga.

Our preferred interpretation for the assembly of Nuna follows that of Kirscher et al.^9,48^. Geological and palaeomagnetic data indicate that assembly was a protracted, multi-stage process involving: (1) the establishment as early as c. 1.8 Ga of a semi-stable Paleoproterozoic connection between proto-Australia/East Antarctica and Laurentia, possibly separated by a narrow marine basin or epicontinental sea, elements of which interacted through shortening and crustal thickening processes (see above), and (2) final reorganisation at c. 1.6 Ga characterised by crustal thickening and high-temperature processes. Despite a lack of evidence for high- and ultrahigh-pressure metamorphism during Paleoproterozoic time, the crustal record from the Nimrod Complex and correlative areas in Australia and North America indicates active metamorphism and magmatism at c. 1.7 Ga that likely reflects an early stage in the history of Nuna. Although there is no evidence in the Nimrod Complex of the activity seen elsewhere at 1.6 Ga, glacial igneous and volcanic clasts sampled from central East Antarctica and Terre Adélie (1.57 and 1.60 Ga, respectively^61,63^) may be an expression of magmatic activity during final Nuna consolidation.

## Conclusions

An interpretation of a two-stage assembly process for Nuna (or at least a protracted assembly^48^) is supported by Palaeoproterozoic and Mesoproterozoic palaeomagnetic data^4,48^ and evidence from the geological records spanning 1.7–1.6 Ga in Australia, East Antarctica, and Laurentia (e.g.,^5,6,23,58,60^). Importantly, such a model is consistent with the results from this study demonstrating the lack of evidence for subduction-related orogenesis between East Antarctica and Laurentia in the Palaeoproterozoic. Our results also highlight the importance of trace-element geochemistry to distinguish different mineral growth processes involved in polyphase formation of crustal rocks. Coupling geochronology and trace-element analysis enabled us to distinguish the disparate igneous and metamorphic stages related to early Nuna supercontinent development and much later Gondwana plate boundary convergence.

## Methods

Laser-ablation inductively-coupled mass spectrometry (LA–ICP–MS) analyses were performed at the University of Adelaide using a RESOlution LR 193 nm Excimer laser and an Agilent 7900 × ICP–MS. Zircon grains from sample 90-131A were separated using traditional mineral separation techniques (heavy-liquid and magnetic procedures), hand-picked, and mounted in an epoxy disk as outlined by Goodge et al*.*^13^. Mounted zircon grains were imaged using back-scatter electron (BSE) and cathodoluminescence (CL) methods at the University of Adelaide with an FEI Quanta MLA–600 scanning electron microscope. The ICP–MS obtained isotopic concentrations from targeted domains in individual zircon grains. Measured concentrations for both unknowns and standards are given in Supplementary Table S1. Zircons were ablated using a 19 μm spot-size. Analyses were conducted with an operating energy of 30 mJ, power output of ~ 2 J/cm^2^, 30% attenuation and a total acquisition time of 60 s, encompassing 30 s of background measurement and 30 s of ablation. The primary reference material used to correct zircon isotopic concentrations was GJ-1 (TIMS ^206^Pb/^238^U: 601.86 ± 0.37 Ma^64^) which gave a weighted-average ^206^Pb/^238^U age of 602.3 ± 2.0 Ma (MSWD = 1.3; n = 24). Reference materials Plešovice (^206^Pb/^238^U: 337.13 ± 0.37 Ma^65^) and 91,500 (^206^Pb/^238^U: 1063.51 ± 0.39 Ma^64^) were used to monitor to accuracy of GJ-1 and returned weighted-average ^206^Pb/^238^U ages of 338.3 ± 1.1 Ma (MSWD = 0.35; n = 24) and 1054.7 ± 4.4 Ma (MSWD = 0.60; n = 24), respectively. The ages for GJ-1 and Plešovice are within uncertainty of published ages^64,65^, and the age for 91,500 is within 1% of the published age^64^. Therefore, the results from the primary and secondary reference materials are considered reliable. Thus, the propagated uncertainty associated with the upper-intercept age from 90-131A zircon (1744 ± 20 Ma) is of reasonable magnitude. The use of a comparatively young primary reference material (GJ-1) to monitor the accuracy of the U–Pb ratios of 90-131A zircons was considered a reasonable approach despite the possibility of larger ^207^Pb/^206^Pb uncertainties (relative to ^207^Pb/^206^Pb uncertainties from an older zircon standard) given the aforementioned reliability of the age of GJ-1, which was tested by determining the ages of the secondary reference materials. Trace-element concentrations were calibrated to the synthetic glass standard, NIST-610^66^. Time-resolved mass spectra were corrected for mass bias and elemental fractionation using Iolite^67^. The data reduction scheme U_Pb_Geochronology4 was used for isotopic concentrations and Trace_Elements_IS was used for trace-element concentrations. Si was used as the internal reference element for trace-element data reduction (Si = 15.32 wt% assuming a stoichiometry of 32.77 wt% SiO_2_).

## Supplementary Information

Supplementary Information
